# Neuroimaging anomalies in asymptomatic middle cerebral artery steno-occlusive disease with normal-appearing white matter

**DOI:** 10.3389/fneur.2023.1206786

**Published:** 2023-08-24

**Authors:** Zhaodi Huang, Xiaona Xia, Shuai Guan, Gaolang Gong, Yishan Luo, Lin Shi, Juntao Zhang, Xiangshui Meng

**Affiliations:** ^1^Department of Radiology, Qilu Hospital (Qingdao), Cheeloo College of Medicine, Shandong University, Qingdao, China; ^2^State Key Laboratory of Cognitive Neuroscience and Learning & IDG/McGovern Institute for Brain Research, Beijing Normal University, Beijing, China; ^3^BrainNow Research Institute, Shenzhen, Guangdong, China; ^4^Department of Imaging and Interventional Radiology, The Chinese University of Hong Kong, Shatin, Hong Kong SAR, China; ^5^GE Healthcare, Precision Health Institution, Shanghai, China

**Keywords:** cortical thickness, diffusion tensor imaging, functional connectivity, functional magnetic resonance imaging, middle cerebral artery

## Abstract

**Background:**

Asymptomatic chronic cerebrovascular steno-occlusive disease is common, but the cognitive function and alterations in the brain’s structural and functional profiles have not been well studied. This study aimed to reveal whether and how patients with asymptomatic middle cerebral artery (MCA) steno-occlusive disease and normal-appearing white matter differ in brain structural and functional profiles from normal controls and their correlations with cognitive function.

**Methods:**

In all, 26 patients with asymptomatic MCA steno-occlusive disease and 22 healthy controls were compared for neurobehavioral assessments, brain volume, cortical thickness, fiber connectivity density (FiCD) value, and resting-state functional connectivity (FC) using multimodal MRI. We also investigated the associations between abnormal cortical thicknesses, FiCD values, and functional connectivities with the neurobehavioral assessments.

**Results:**

Patients performed worse on memory tasks (Auditory Verbal Learning Test-Huashan version) compared with healthy controls. Patients were divided into two groups: the right group (patients with right MCA steno-occlusive disease) and the left group (patients with left MCA steno-occlusive disease). The left group showed significant cortical thinning in the left superior parietal lobule, while the right group showed significant cortical thinning in the right superior parietal lobule and caudal portion of the right middle frontal gyrus. Increased FiCD values in the superior frontal region of the left hemisphere were observed in the left group. In addition, a set of interhemispheric and intrahemispheric FC showed a significant decrease or increase in both the left and right groups. Many functional connectivity profiles were positively correlated with cognitive scores. No correlation was found between cortical thickness, FiCD values, and cognitive scores.

**Conclusion:**

Even if the patients with MCA steno-occlusive disease were asymptomatic and had normal-appearing white matter, their cognitive function and structural and functional profiles had changed, especially the FC. Alterations in FC may be an important mechanism underlying the neurodegenerative process in patients with asymptomatic MCA steno-occlusive disease before structural changes occur, so FC assessment may promote the detection of network alterations, which may be used as a biomarker of disease progression and therapeutic efficacy evaluation in these patients.

## Introduction

Chronic carotid or cerebral artery severe (>70%) stenosis or occlusion disease are the common causes of chronic cerebral hypoperfusion (CCH) (1–5), which has been positively identified as a key mechanism leading to vascular cognitive impairment and dementia (2, 6–9). Many studies have shown the changes in the structure, brain network, cognitive function, and their relationships in patients with chronic carotid artery severe stenosis or occlusion disease (10–16). One study showed neither a clear pattern of reduced cortical thickness nor an association of cortical thickness with cognitive function in patients with severe asymptomatic carotid stenosis (SACS) (17). These patients can be clinically asymptomatic or symptomatic. Symptoms include sensorimotor, pure sensory, and cognitive impairment and depression. Furthermore, patients with subtle cognitive impairment (including attention, memory, processing speed, and executive function) may be neglected due to normal-appearing white matter in general MRI and little attention in the clinical setting. However, the cognitive impairment can gradually progress, and finally lead to subsequent dementia (3). However, the underlying pathophysiology remains unclear and is multifactorial.

Despite promising results revealing the changes in the structure and network in patients with severe asymptomatic carotid stenosis, studies have some shortcomings. A major limitation is that current diffusion tensor imaging (DTI) studies cannot provide a direct comprehensive analysis of the structural connectivity of the whole cortex. A recent technique named fiber connectivity density (FiCD) mapping was proposed to map the structural connectivity properties (e.g., fractional anisotropy, or mean diffusivity) to the cortical surface, to create a common space for comparison with results from brain functional analysis and cortical thickness analysis, which can automatically identify brain regions with significant differences at the whole-brain level without defining ROIs (18, 19). Studies had shown that FiCD values were significantly changed in cerebral small-vessel disease (CSVD) patients (20) with mild cognitive impairment (MCI) and in patients with end-stage renal disease (ESRD) (21). FC can reveal brain activity, which has become an important tool in the study of neurological diseases (22), and numerous studies proved that decreased FC was related to impaired cognition in various diseases.

For another aspect, diffusion and metabolic changes in regions of cerebral artery obstruction in patients with symptomatic MCA steno-occlusive disease (defined as >70% stenosis on maximum intensity projection images or a complete signal loss of the middle cerebral artery trunk on magnetic resonance angiography) and normal-appearing white matter have been revealed (23, 24). Nonetheless, structural changes, FiCD values, FC, and the relationships between them and cognitive function in these patients have not been investigated, which would likely provide more pathophysiological information that can lead to a better understanding of the mechanisms of cognitive impairment in these patients.

In this study, we recruited a group of patients with asymptomatic MCA steno-occlusive disease and normal-appearing white matter. Multimodal MRI (structural, diffusion, and resting-state functional MRI) measures were compared for brain volume, cortical thickness, fiber connectivity density (FiCD) value, and functional connectivity (FC) between patients and an age-, sex-, and education-matched healthy control (NC) group. The associations between abnormal cortical thicknesses, FiCD values, and functional connectivities with the neurobehavioral assessments were performed.

## Materials and methods

### Participants

A total of 26 patients were recruited between January 2017 and June 2021 and were divided into two groups, namely, the right group (patients with right MCA steno-occlusive disease) and the left group (patients with left MCA steno-occlusive disease), as discovered by Magnetic Resonance Angiography (MRA). The inclusion criteria were as follows: (a) unilateral MCA stenosis >70%, (b) free of stroke, TIA, or dementia, (c) right-handed, (d) capable of completing the MRI examination with a qualifying high-resolution MRI image, (e) no history of drug use that could affect cognitive function, and (f) normal-appearing white matter (apparently normal brain parenchymal signals or lacunar infarcts<3 mm in diameter on T2-weighted and FLAIR sequences). The exclusion criteria were as follows: (a) any other cerebral arteries stenosis ⩾30%, (b) a history of severe systemic diseases and neuropsychiatric diseases, (c) a history of frequent dizziness and headache, (d) a history of acute or chronic cerebral infarction, bleeding, tumor, infectious disease, or metabolic disease detected by MRI, (e) a history of drug or alcohol dependence during the last 6 months, and (f) contraindications for MRI. A total of 22 age-, sex-, and education-matched healthy subjects were recruited from the community as an NC group. The inclusion criteria for the control group were as follows: MMSE score ≥ 27 and ADL score = 14, with other criteria being the same as the inclusion criteria of the patient group. The exclusion criteria were as follows: (a) White matter hyperintensity or/and lacunar infarcts ≥3 mm in diameter and (b) any cerebral artery stenosis ⩾30%; with other criteria being the same as (b) ~ (f) of the exclusion criteria of the patient group. The present study was approved by the Medical Ethics Committee of the Qilu Hospital (Qingdao) of Shandong University and informed consent was obtained for all participants.

Detailed demographic characteristics of the subjects are listed in Table 1.

**Table 1:** The demographic characteristics of the subjects.

	NC (*n* = 22)	Patient group		NC *vs* Left	NC *vs* Right
		Left (*n* = 14)	Right (*n* = 12)	z/t(d)	*p*-value	z/t(d)	*p*-value
Age (years, median, IQR)	61.0 (11.00)	64.5 (13.25)	59.0 (11.50)	1.764	0.083^a^	0.489	0.631^a^
Sex (female, %)	11 (50.00%)	8 (57.14%)	5 (41.67%)	*N*	0.742^b^	*N*	0.729^b^
Education (years, mean, SD)	9.3 (2.98)	9.4 (3.28)	9.4 (4.14)	−0.175 (−0.032)	0.862^c^	0.106(−0.028)	0.916^c^
Hypertension (%)	7 (31.82%)	10 (71.43%)	8 (66.67%)	*N*	**0.039** ^**b**^	*N*	0.075^b^
Diabetes (%)	3 (13.64%)	8 (57.14%)	3 (25.00%)	*N*	**0.010** ^**b**^	*N*	0.641^b^
Hyperlipidemia (%)	5 (22.73%)	7 (50.00%)	5 (41.67%)	*N*	0.148^b^	*N*	0.271^b^
Current smoker (%)	1 (4.54%)	3 (21.43%)	5 (41.67%)	*N*	0.277^b^	*N*	**0.014** ^**b**^
Current drinker (%)	8 (36.36%)	2 (14.29%)	4 (33.33%)	*N*	0.255^b^	*N*	1.000^b^

a Mann–Whitney U test.

b Fisher’s exact test.

c Independent-samples t-test.

*p*-value significant cut-off 0.05.

IQR, Interquartile range; SD, Standard deviation; d, Cohen’s d; *N*, no *χ*^2^.

Bold values: *p*-value < 0.05.

### Neurobehavioral assessments

Neurobehavioral assessments were performed within 2 days of MRI scanning, including the Mini-mental State Examination (MMSE) (25), which was used to assess global cognition. Language proficiency, processing speed, cognitive flexibility, verbal learning/memory, and activities of daily living were assessed, respectively, with the Boston Naming Test (BNT) (9), Trail-Making Test (TMT) A and B (9), Auditory Verbal Learning Test-Huashan version (AVLT-H) (26), and Activity of Daily Living Scale (ADL) (27).

### MRI data acquisition

All data were collected using a 3T MRI scanner (Ingenia, Philips Medical Systems, Netherlands). The matched head coil with foam padding and earplugs was used to reduce head motion and scanner noise. The scanning sessions included: (1) T2WI (TR/TE = 2369/107 ms, matrix = 352 × 352, 18 axial slices, 6-mm slice thickness with a 1-mm gap), (2) (T2WI-FLAIR) (TR/TE = 7000/125 ms, matrix = 288 × 163, 18 axial slices, 6-mm slice thickness with a 1-mm gap), (3) DWI (TR/TE = 2235/76 ms, matrix = 176 × 134, 18 axial slices, 6-mm slice thickness with a 1-mm gap), (4) three-dimensional T1-weighted imaging (3D-T1WI) (TR/TE = 6.7/3.0 ms, 170 sagittal slices, 1-mm slice thickness with no gap), (5) DTI (TR/TE = 4900/95 ms, matrix = 122 × 110, 70 axial slices, 2 mm slice thickness with no gap, b values =1000s/mm^2^) in 32 directions, and (6) resting state functional magnetic resonance imaging (r-fMRI) (TR/TE = 2000/30 ms, FA = 90°, FOV = 230 × 230 mm^2^, data matrix = 68 × 66, Voxel = 3.5 × 3.5 × 4 mm^3^, 32 axial slices, 4-mm slice thickness with a 0.5-mm gap, 240 time points).

### MRI data processing

3D-T1WI images were processed using AccuBrain® IV2.0 (Brainnow Medical Company Limited, Shenzhen, PR China), a brain quantification tool that performs brain structure and tissue volume quantification in a fully automatic mode. Its segmentation accuracy has been validated (28). AccuBrain^®^ IV2.0 provides 66 quantitative brain regional volumetric indexes including the volumes of the hippocampus, lateral ventricle, amygdala, etc. It also divided the cerebral cortex into 12 lobular regions, including the frontal lobe (L/R), temporal lobe (L/R), parietal lobe (L/R), occipital lobe (L/R), cingulate lobe (L/R), and insular lobe (L/R); and the atrophy of each was measured as the ratio of the CSF volume to the parenchyma volume within that lobular region.

To perform cortical thickness analysis, 3D-T1WI images were also processed using FreeSurfer20 (29) with the automatic “recon-all” pipeline. The cortical thickness was measured individually and compared between groups.

DTI data were processed using the FiCD pipeline (19). The FiCD mapping method was based on a combination of a diffusion fiber tracking technique and cortical surface-based analysis (30, 31). First, the GM-WM interface was extracted and parcellated into 1,000 cortical units (CUs). Second, the WM tractography was constructed, and the CUs were transformed into tractography space. Third, association fibers (AFs) were tracked and the FiCD value was calculated for each CU, then the whole-cortex FiCD map was generated. Fourth, the FiCD map in volume space was projected onto the cortical surface, then registered to the standard brain surface and smoothed with Gaussian kernels at a series of full width at half-maximum (FWHM). Preprocessing of the diffusion data was performed using the FSL (PANDA) toolbox (32), whereas preprocessing of the 3D-T1WI images was performed using the Freesurfer software.

In addition to structural image analysis, r-fMRI data was also analyzed using the CONN toolbox (33). It first preprocessed the r-fMRI and 3D-T1WI images using a standard pipeline, including susceptibility distortion correction, motion correction/realignment, slice-timing correction, outlier identification, coregistration, tissue-class segmentation, MNI-normalization, and smoothing. Moreover, r-fMRI data were denoised using the regression of confounding factors characterized by white matter time series, cerebrospinal fluid time series, motion parameters, and linear trends within each function run. Temporal filtering with a band of 0.009–0.08 Hz was conducted to the time series of each voxel to reduce the impact of low-frequency drifts and high-frequency noise.

Seed-based correlation (SBC) and region-of-interest to region-of-interest (ROI-to-ROI) analysis were performed. The ROIs were derived from the Harvard-Oxford Atlas, including 132 ROIs of the whole brain, e.g., frontal pole right (FP r), frontal pole left (FP l), insular cortex right (IC r), and insular cortex left (IC l). Group-level statistical analysis was implemented using correlation analysis. A standard second-level General Linear Model analysis of r-fMRI connectivity matrices produced a single statistical matrix of T- or F- values, characterizing the effect of interest (e.g., the difference in connectivity between two groups) among all possible pairs of ROIs.

**Figure 1:**
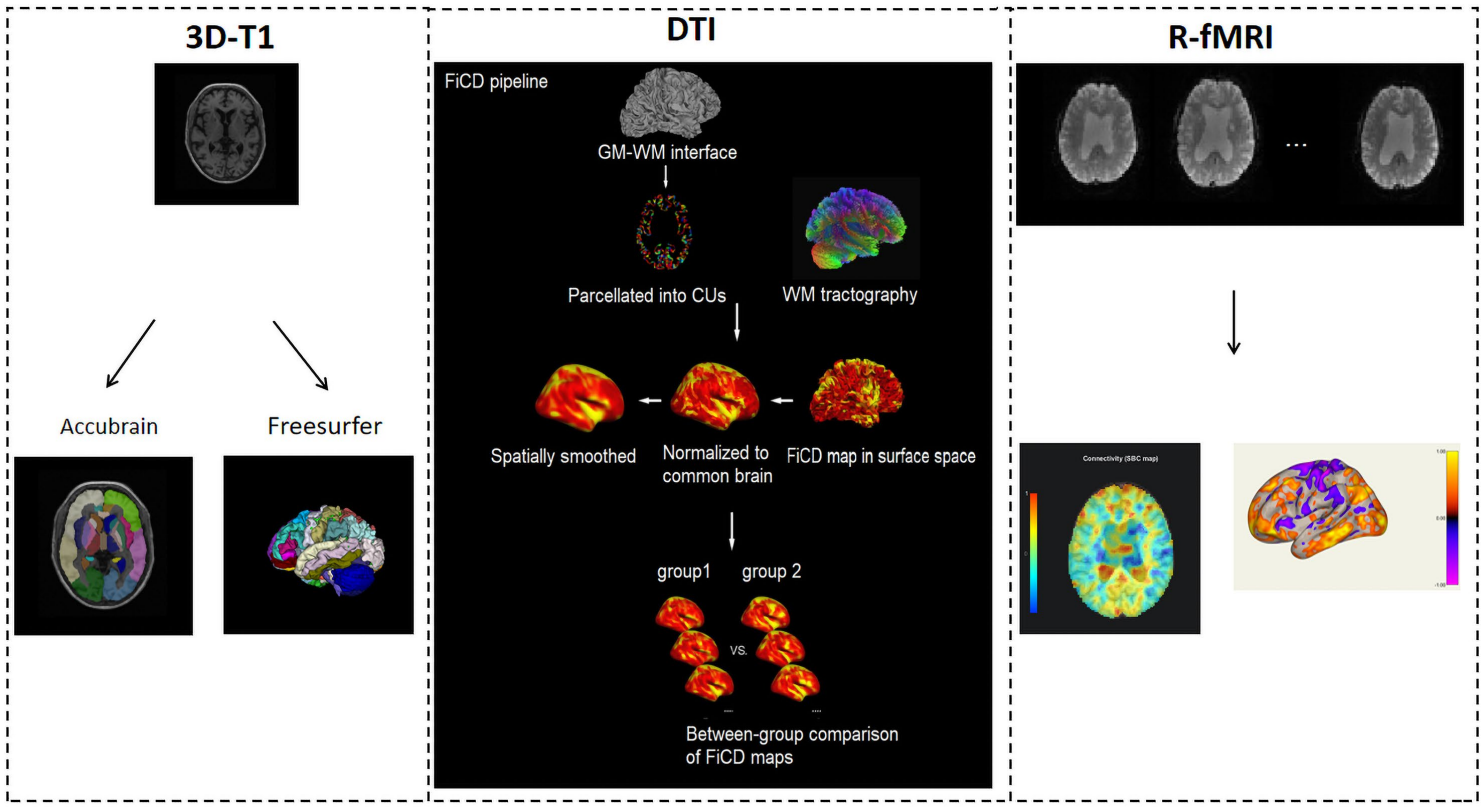
MRI data processing pipeline.

The overall data processing pipeline is shown in Figure 1.

### Statistical analysis

Fisher’s exact test, independent-samples t-test, and Mann–Whitney U test were performed to determine if there were statistically significant differences between groups. Statistical analysis of demographic characteristics and neurobehavioral assessment results were performed using the Statistical Package for the Social Sciences, version 25 (SPSS 25, Chicago, Illinois) with a significance level set at *p* < 0.05.

The Mann–Whitney U test was used to compare differences in brain structural volume quantification between groups. The results were reported with false discovery rate (FDR) multiple-comparison corrections. FDR corrected *p* < 0.05 was considered statistically significant.

For r-fMRI, parametric multivariate statistics (cluster-level inferences) were reported, with cluster-level p-FDR corrected threshold set as *p* < 0.05.

The statistical analysis on surface-based results, i.e., cortical thickness and FiCD, was processed using Qdec included in the Freesurfer toolbox. It was intended to perform inter-group averaging and inference on the morphometry data (cortical surface and volume) produced by the FreeSurfer processing stream. For each hemisphere, the General Linear Model (GLM) was computed vertex-by-vertex to analyze cortical FiCD values. The cortical maps were smoothed using a 10 mm FWHM Gaussian kernel before comparison to reduce the noise caused by spatial normalization. A significant threshold of *p* < 0.05 was adopted (Monte Carlo Null-Z simulation was used to correct for multiple comparisons).

Pearson and Spearman correlation analysis was used to assess the relationships between neurobehavioral assessment results with cortical thickness, FiCD values, and FC. Then, multifactor linear regression analysis controlling confounding factors such as hypertension and smoking was performed to investigate whether the correlations were affected by confounding factors.

## Results

### Neurobehavioral assessment results

The scores of AVLT-H (immediate, short-delayed, and long-delayed) in the left group and AVLT-H (immediate and long-delayed) in the right group were significantly lower compared to healthy controls. The patient groups did not show a significant decline in global cognition and other multidomain neurobehavioral assessments. Detailed neurobehavioral assessment results of the subjects are listed in Table 2. The ADL scores of the left group and the right group were 14.

**Table 2:** The neurobehavioral assessment results of the subjects.

	NC (*n* = 22)	Patient Group		NC *vs* Left		NC *vs* Right	
		Left (*n* = 14)	Right (*n* = 12)	z/t(d)	*p*-value	z/t(d)	*p*-value
MMSE (median, IQR)	28.0 (1.25)	28.0 (3.25)	28.0 (2.75)	−1.283	0.215^a^	−1.594	0.127^a^
BNT (median, IQR)	25.5 (3.50)	26.5 (4.75)	28.0 (4.75)	1.176	0.253^a^	1.324	0.191^a^
TMT-A (median, IQR)	40.0 (24.75)	44.0 (31.25)	50.0 (31.00)	0.715	0.490^a^	0.667	0.511^a^
TMT-B (median, IQR)	50.5 (27.00)	72.5 (43.75)	73.0 (42.25)	1.948	0.053^a^	0.955	0.345^a^
AVLT-H, immediate (mean, SD)	18.5 (3.62)	14.6 (5.56)	15.3 (4.70)	2.575 (0.831)	**0.015** ^**b**^	2.193 (0.763)	**0.036** ^**b**^
AVLT-H, short delayed (5 min), (mean, SD)	6.7 (2.21)	4.9 (2.54)	5.1 (2.84)	2.338 (0.756)	**0.025**	1.874 (0.629)	0.070^b^
AVLT-H, long delayed (20 min), (mean, SD)	6.6 (2.02)	4.0 (3.44)	4.7 (2.81)	2.597 (0.922)	**0.018** ^**b**^	2.369 (0.776)	**0.024** ^**b**^

a Mann–Whitney U test.

b Independent-samples t-test.

*p*-value significant cut-off 0.05.

IQR, Interquartile range; SD, standard deviation; MMSE, Mini-mental state examination; BNT, Boston naming test; TMT-A/B, Trail-making test A and B; AVLT-H, Auditory Verbal Learning Test-Huashan version; d, Cohen’s d.

Bold values: *p*-value < 0.05.

### Structural volumetric results

Brain structural volume quantification results showed no significant difference after FDR correction between the left group and NC group, and the right group and NC group.

### Cortical thickness results

**Figure 2:**
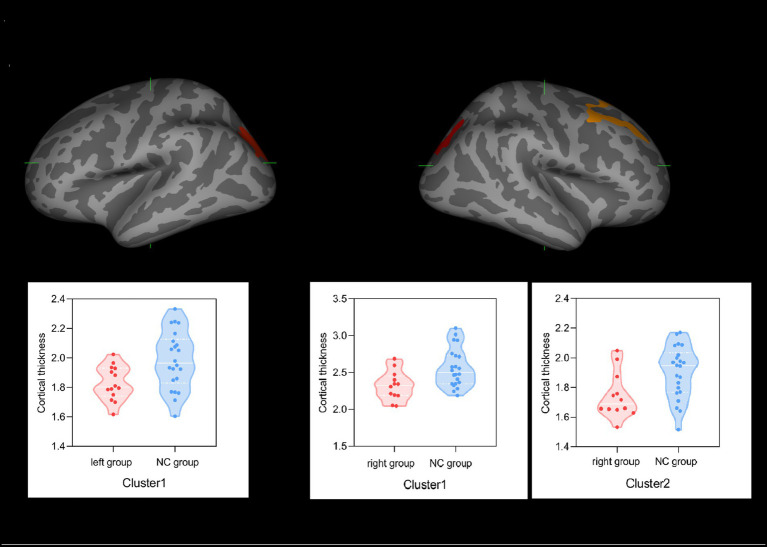
Group effect on cortical thicknesses. **(A)** Left superior parietal lobule (cluster 1) in the left group showed significant thinning of cortical thickness compared with the NC group. **(B)** Caudal portion of the right middle frontal gyrus (cluster 1) and right superior parietal lobule (cluster 2) regions in the right group showed significant thinning of cortical thickness compared with the NC group.

In terms of cortical thickness measurement, as shown in Figure 2, in the left group and NC group comparison, the left group showed significant cortical thinning of the left superior parietal lobule (cluster 1: *p* = 0.0104), while in the right group and NC group comparison, the right group showed significant cortical thinning of the caudal portion of the right middle frontal gyrus (cluster 1: *p* = 0.0003) and superior parietal lobule (cluster 2: *p* = 0.0357). No correlation was found between cortical thickness and AVLT-H scores.

### FiCD results

**Figure 3:**
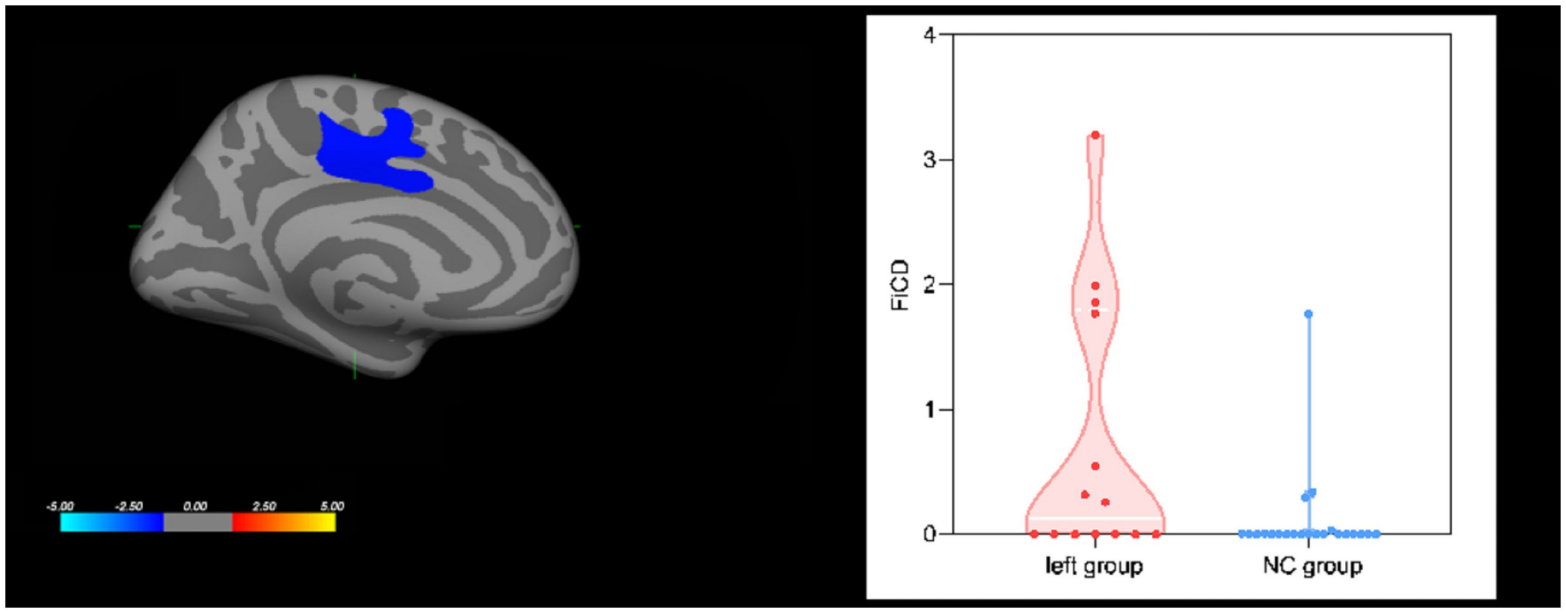
Group effect on cortical FiCD values. The left superior frontal region showed increased FiCD values in the left group compared with the NC group.

With FiCD measurement, FiCD values increased in the left superior frontal region (cluster 1: *p* = 0.0279) in the left group compared with the NC group, as shown in Figure 3, while there was no significant difference between the right group and the NC group. No correlation was found between FiCD values and AVLT-H scores.

### Functional connectivity

**Figure 4:**
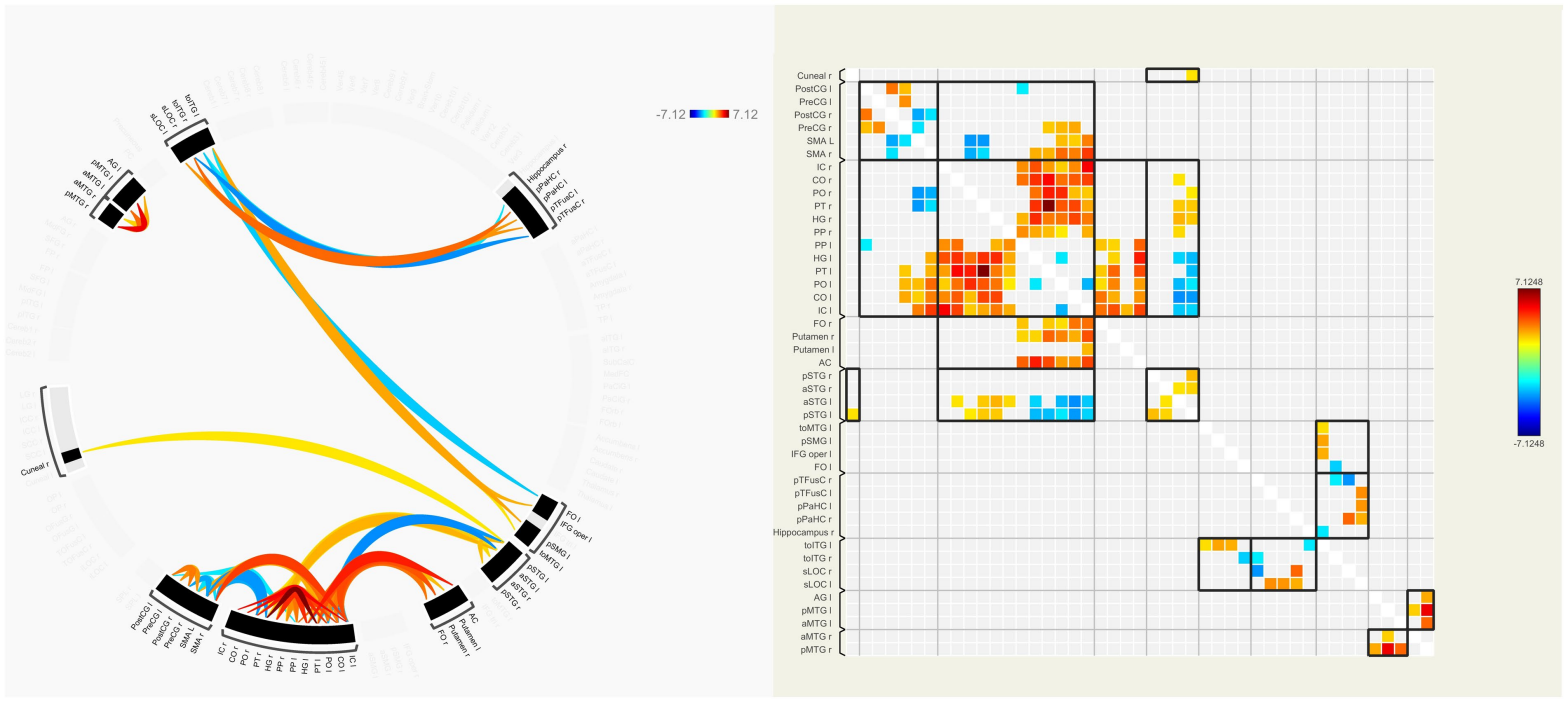
ROI-to-ROI functional connectivities between the left group and the NC group. The decreased (orange) and increased (blue) functional connectivities in left MCA steno-occlusive patients after FDR correction are shown. Patients showed more significantly decreased functional connectivities in the left and right parts of the regions such as PT, PO, CO, IC, HG, MTG, and CG, while increased connectivities in the left and right parts of SMA, between the right part of PO, PT, and SMA, and between the left part of STG and HG, PT, PO, CO, and IC. ROI-to-ROI, region-of-interest to region-of-interest; NC, normal control; MCA, Middle cerebral artery; FDR, False discovery rate; PT, Planum Temporale; PO, Parietal Operculum Cortex; CO, Central Opercular Cortex; IC, Insular Cortex; HG, Heschl’s Gyrus; MTG, Middle Temporal Gyrus; CG, Cingulate Gyrus; SMA, formerly Supplementary Motor Cortex; STG, Superior Temporal Gyrus.

**Figure 5:**
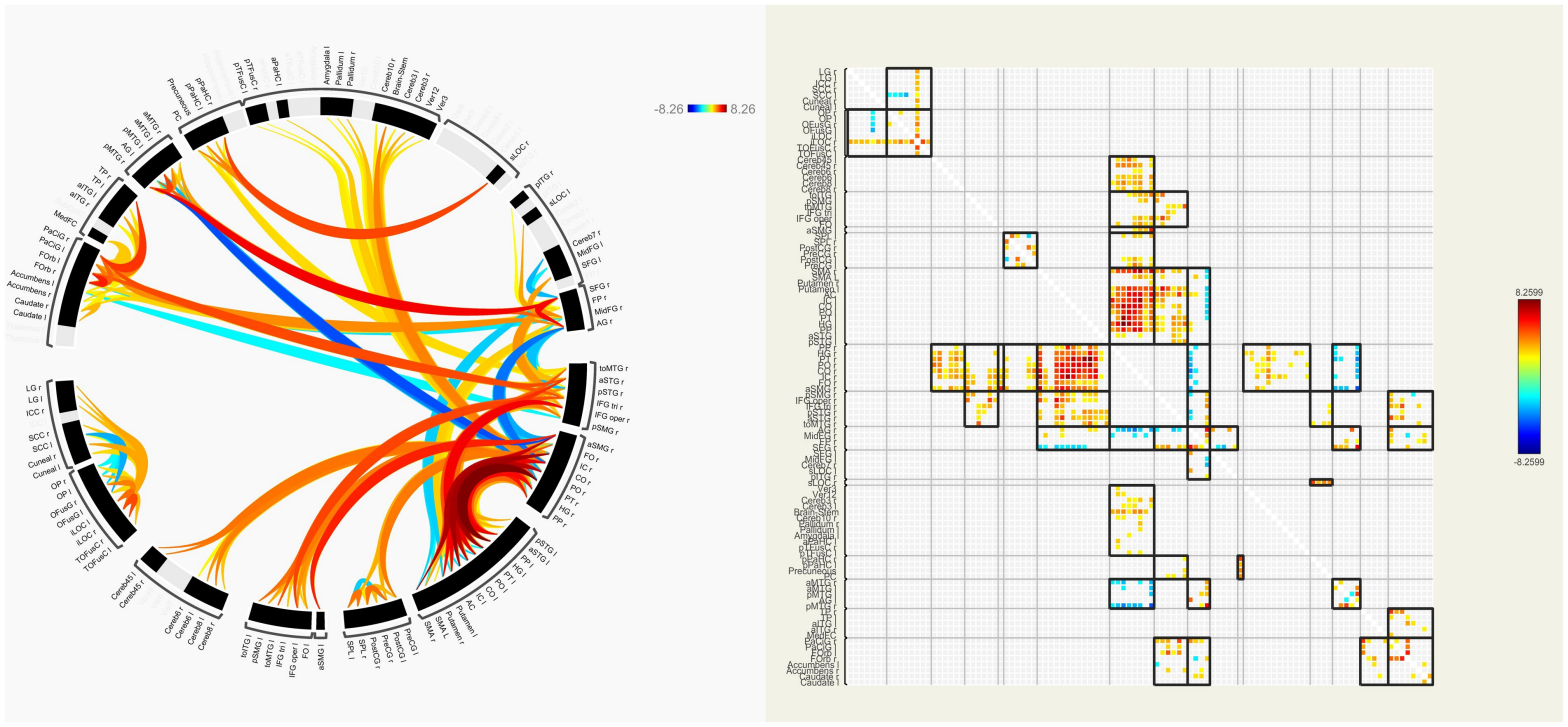
ROI-to-ROI functional connectivities between the right group and the NC group. The decreased (orange) and increased (blue) functional connectivities in right MCA steno-occlusive patients after FDR correction are shown. Patients showed more significant decreased functional connectivities in the left and right parts, e.g., PT, PO, CO, IC, CG, HG, PP, FO, and FOrb, while showing increased connectivity between the right AG and HG, PT, PO, CO, IC, and SMG, and between the right MTG and PP, HG, PT, PO, CO, IC, and SMG. ROI-to-ROI, region-of-interest to region-of-interest; NC, normal control; MCA, Middle cerebral artery; FDR, False discovery rate; PT, Planum Temporale; PO, Parietal Operculum Cortex; CO, Central Opercular Cortex; IC, Insular Cortex; CG, Cingulate Gyrus; HG, Heschl’s Gyrus; PP, Planum Polar; FO, Frontal Operculum Cortex; FOrb, Frontal Orbital Cortex; AG, Angular Gyrus; SMG, Supramarginal Gyrus, MTG, Middle Temporal Gyrus.

In our patients, FC of many interhemispheric and intrahemispheric regions showed changes in the left and right groups. Compared with the NC group, 71 interhemispheric and 10 intrahemispheric (including 9 of the left hemisphere and 1 of the right hemisphere) functional connectivity profiles significantly decreased and 6 interhemispheric and 17 intrahemispheric (including 12 of the left hemisphere and 5 of the right hemisphere) functional connectivity profiles significantly increased (FDR corrected, *p* < 0.05) in the left group. There were 230 interhemispheric and 75 intrahemispheric functional connectivity profiles that significantly decreased and 14 interhemispheric and 30 intrahemispheric (including 28 of the right hemisphere and 2 of the left hemisphere) functional connectivity profiles that significantly increased (FDR corrected, *p* < 0.05) in the right group. The results of functional connectivity between different groups are shown in Figures 4, 5. We found 5 functional connectivity profiles were correlated with AVLT-H, immediate scores, 3 functional connectivity profiles were correlated with AVLT-H, short delayed scores, and 7 functional connectivity profiles were correlated with AVLT-H, long-delayed scores in the left group. In the right group, there were 19 functional connectivity profiles that correlated with AVLT-H, immediate scores, and 7 functional connectivity profiles that correlated with AVLT-H, long-delayed scores. Then, multifactor linear regression analysis controlling confounding factors was performed, discovering 3 functional connectivity profiles correlated with AVLT-H, immediate scores, 2 functional connectivity profiles correlated with AVLT-H, short-delayed scores, and 3 functional connectivity profiles correlated with AVLT-H, long-delayed scores in the left group. The detailed results are listed in Table 3. In the right group, there were 18 functional connectivity profiles that correlated with AVLT-H, immediate scores, and 6 functional connectivity profiles that correlated with AVLT-H, long-delayed scores. The detailed results are listed in Table 4.

**Table 3:** ROI-to-ROI functional connectivities correlated with AVLT-H scores in the left group.

	ROI-to-ROI	B (95% CI)	*p*-value
AVLT-H, immediate	IC r-pp l	22.244 (4.020 ~ 40.468)	0.022
	CO r-aSTG l	15.350 (1.506 ~ 29.194)	0.033
	pSTG r-pSTGl	19.081 (0.769 ~ 37.393)	0.043
AVLT-H, short delayed (5 min)	pSTG r-pSTG l	13.028 (7.296 ~ 18.760)	0.006
	IFG oper l-to lTG l	12.999 (3.525 ~ 22.474)	0.012
AVLT-H, long delayed (20 min)	pSTG r-pSTG l	14.555 (1.662 ~ 19.006)	0.011
	CO r-aSTG l	10.334 (4.107 ~ 25.007)	0.024
	IFG oper l-to ITG l	14.658 (0.125 ~ 29.129)	0.048

ROI-to-ROI, region-of-interest to region-of-interest; CI, confidence interval; AVLT-H, Auditory Verbal Learning Test-Huashan version; ICr, Insular Cortex Right; PPl, Planum Polar Left; COr, Central Opercular Cortex Right; aSTGl, Superior Temporal Gyrus, anterior division Left; pSTGr, Superior Temporal Gyrus, posterior division Right; pSTGl, Superior Temporal Gyrus, posterior division Left; IFG operl, Inferior Frontal Gyrus, pars opercularis Left; toITGl, Inferior Temporal Gyrus, temporooccipital part Left.

**Table 4:** ROI-to-ROI functional connectivities correlated with AVLT-H scores in the right group.

	ROI-to-ROI	B (95% CI)	*p*-value
AVLT-H, immediate	PT r-PT l	23.563 (16.084 ~ 31.043)	0.000
	HG r-Putamen l	23.479 (10.771 ~ 36.186)	0.002
	CO r-HGl	17.224 (7.304 ~ 27.145)	0.003
	PO r-Putamen l	17.224 (2.272 ~ 34.610)	0.003
	PT r-HG l	19.501 (8.230 ~ 30.771)	0.004
	IC r-HG l	15.626 (6.478 ~ 24.775)	0.004
	pMTG l-AG l	16.847 (6.985 ~ 26.709)	0.004
	PostCG r-Putamen l	18.823 (7.715 ~ 29.931)	0.004
	PP r-CO l	24.339 (9.039 ~ 39/638)	0.006
	sLOC r-pPaHC r	34.313 (10.820 ~ 57.805)	0.009
	PP r-IC l	21.765 (6.724 ~ 36.807)	0.010
	PT r-Putamen l	14.260 (3.517 ~ 25.003)	0.015
	PreCG r-Putamen l	17.537 (3.545 ~ 31.530)	0.020
	PP r-MidFG r	32.222 (6.326 ~ 58.114)	0.020
	IC r-PT l	17.723 (3.429 ~ 32.017)	0.021
	PT r-AC	11.767 (1.448 ~ 22.087)	0.030
	PP r-HG l	15.232 (1.042 ~ 29.422)	0.038
	CO r-Putamen l	17.683 (0.706 ~ 34.660)	0.043
AVLT-H, long delayed (20 min)	HG r-AC	10.816 (1.468 ~ 20.163)	0.028
	sLOC r-pPaHC r	18.120 (2.499 ~ 33.740)	0.028
	HG r-Putamen l	11.164 (1.336 ~ 20.993)	0.030
	PT r-AC	6.770 (0.504 ~ 13.036)	0.037
	PO r-Putamen l	10.504 (0.623 ~ 20.385)	0.040
	IFGtri r-PaCiG l	9.844 (0.479 ~ 19.209)	0.041

ROI-to-ROI, region-of-interest to region-of-interest; CI, confidence interval, AVLT-H, Auditory Verbal Learning Test-Huashan version; PTr, Planum Temporale Right; PTl, Planum Temporale Left; HGr, Heschl’s Gyrus Right; Putamen l, Putamen Left; COr, Central Opercular Cortex Right; HGl, Heschl’s Gyrus Left; ICr, Insular Cortex Right; pMTGl, Middle Temporal Gyrus, posterior division Left; AGl, Angular Gyrus Left; PostCGr, Postcentral Gyrus Right; PPr, Planum Polar Right; COl, Central Opercular Cortex Left; sLOCr, Lateral Occipital Cortex, superior division Right; pPaHCr, Parahippocampal Gyrus, posterior division Right; ICl, Insular Cortex Left; PreCGr, Precentral Gyrus Right; MidFGr, Middle Frontal Gyrus Right; AC, Cingulate Gyrus, anterior division; HGr, Heschl’s Gyrus Right; sLOCr, Lateral Occipital Cortex, superior division Right; IFGtrir, Inferior Frontal Gyrus, pars triangularis Right; PaCiGl, Paracingulate Gyrus Left.

## Discussions

This study systematically conducted a cross-sectional neuroimaging analysis of asymptomatic MCA steno-occlusive disease patients with NAWM to increase knowledge about the possible changes in brain volume, cortical thickness, FiCD value, FC, and cognition associated with this condition. Our study focused on the main effects of groups than on the differences between the left and right sides of the brain on account of the effect of the dominant hemisphere.

We found that brain structural volume quantification showed no difference among the left group, right group, and NC group. Nevertheless, the left group showed significant thinning of the left superior parietal lobule, while the right group showed significant thinning of the right superior parietal lobule and caudal portion of the right middle frontal gyrus in cortical thickness. This phenomenon may be caused by hypoperfusion and different susceptibility to hypoperfusion. First, with respect to hypoperfusion, previous investigations confirmed that patients with the carotid steno-occlusive disease had an increased risk of cerebral hypoperfusion (3, 4, 16, 34–36), which, gradually, may lead to cerebral atrophy (11, 14, 37, 38). Second, Marshall et al. (16) found that even if the posterior circulation regions also showed hypoperfusion in vertebrobasilar disease, the occipital and cerebellar cortices were barely affected, which led them to speculate that the anterior circulation region, but not the posterior circulation, is susceptible to hypoperfusion. Moreover, we only found two regions with decreased cortical thickness in our study, which may reflect the different susceptibility to hypoperfusion in different cerebral regions and even the same regions in different hemispheres. Ordinarily, neurodegenerative diseases tend to begin with a single vulnerable brain region, followed by trans-synaptic spread (39). Therefore, further longitudinal follow-up studies are needed. Studies have shown that atrophy of different regions in patients with CAS may result in different kinds of cognitive impairments (2, 14, 37, 38) and plenty of studies had reported memory decline in patients with CAS (40, 41). In our study, patient groups had lower scores in AVLT-H, suggesting memory decline, whereas no correlations were found between cortical thickness and AVLT-H scores. It may be related to a small sample size, or our neurobehavioral assessments could not discover their subtle cognitive impairments related to these regions with cortical thickness thinning, which needs further study.

We did not find any decreased FiCD value in the left and right groups. We interpreted the lack of decrease in FiCD value as an indicator of the robustness of the white matter to CCH, which may benefit from the lower oxygen demand compared with gray matter. Furthermore, compensatory circulation and neurovascular coupling may help maintain white matter’s structural and functional metabolism. Besides, the left group showed increased FiCD values in the superior frontal region of the left hemisphere, which reflected the increased fibrous connections in this region. This may be a compensatory mechanism in the brain that has not been reported previously. Otherwise, we were not sure whether it was caused by a small sample size, other confounding factors, or an analytical method in our study. Thus, further studies are needed to evaluate this speculation.

With regard to FC, we observed that functional connectivity of many interhemispheric and intrahemispheric regions showed decrements, especially between interhemispheric regions and intrahemispheric regions of the stenotic side in the left and right groups. Compatible with our current findings, one article about patients with asymptomatic MCA steno-occlusive disease indicated significant decreases in network strength, global efficiency, and the clustering coefficient, as well as a longer characteristic path length (42). We found many functional connectivity profiles were positively correlated with AVLT-H scores, thus we speculated that the subtle cognitive impairment may be attributed to the combined effect of the decreased FC profiles. Many studies showing decreased functional connectivity and increased risk of cognitive fragility in patients with SACS (12, 13, 15, 36, 43, 44) confirmed our speculation. Moreover, we found hyper-connectivity in many brain regions between the ipsilateral and bilateral hemispheres of patients in our study, reflecting compensatory effects for maintaining better cognitive function. Studies had reported compensatory hyper-connectivity in the healthy hemisphere and a few hyper-connectivity in the stenotic sides of patients with SACS (45, 46). Thus, we speculated that hyper-connectivity may be an important mechanism that maintains clinical asymptomatic performance and better cognition function. In addition, the collateral network (primary and secondary) may be another compensatory mechanism, which had not been discovered in our study and needs further research. Furthermore, we found that the decrement of FC in the right group was much more diffuse than that in the left group, which may also reflect the different susceptibility to hypoperfusion in different cerebral regions. The effect of the dominant hemisphere may be another explanation because we only recruited right-handed patients, therefore, further studies involving left-handed patients are needed to evaluate this speculation. Otherwise, one study also showed that part of the FC alterations gradually recovered to the normal condition after carotid artery stenting in patients with carotid stenosis (46). So, FC may be used as a biomarker of disease progression and therapeutic efficacy evaluation in these patients’ chronic cerebrovascular steno-occlusive disease. Notably, it was difficult to directly compare these studies because the functional measures differed.

In the present study, we found numerous regions with decreased FC in regions without declined cortical thickness and FiCD value, thus, we speculated that decreases in FC may exist prior to the structural changes including white and gray matter, which represented a higher sensitivity of FC. The alterations in FC may be an important mechanism underlying the neurodegenerative process in patients with carotid or cerebral arterial diseases causing early cognition impairment, which may promote the detection of network alterations, helping provide guidance for early clinical interventions in advance and reduce the impairment of higher brain functions in patients with carotid or cerebral arterial diseases.

There were several limitations in this study. First, our number of patients was small, limiting the statistical ability to identify small effect size variations. Second, our neurobehavioral assessments were relatively simple, and more elaborate neurological scales could be conducted in the future. Third, we did not evaluate the small vessels or microvessels of the patients, because the interval time between perfusion imaging and multimodal MRI was too long. Finally, the acquisition time of r-fMRI in this study was relatively short, and longer scanning time is preferred to provide more robust information on functional connectivity.

## Conclusion

Even if the patients with MCA steno-occlusive disease were asymptomatic and had normal-appearing white matter, their cognitive function, structural and functional profiles had changed, especially the FC. And, alterations in FC in patients with asymptomatic MCA steno-occlusive disease occurred before structural changes. Hyper-connectivity may act as the compensation factor in neuroplasticity, maintaining clinical asymptomatic performance and better cognitive function. So, FC assessment may promote the detection of network alterations, which may be used as a biomarker of disease progression and therapeutic efficacy evaluation in patients with chronic cerebrovascular steno-occlusive disease.

## Data availability statement

The original contributions presented in the study are included in the article/supplementary material, further inquiries can be directed to the corresponding author.

## Ethics statement

The studies involving humans were approved by Medical Ethics Committee of the Qilu Hospital (Qingdao) of Shandong University. The studies were conducted in accordance with the local legislation and institutional requirements. The participants provided their written informed consent to participate in this study.

## Author contributions

XM conceived and designed the research. ZH performed the experiments. ZH, XX, and SG collected the data. ZH, XX, LS, and YL performed the imaging analysis. ZH and XX wrote, reviewed, and edited the manuscript. GG, JZ, and LS reviewed and edited the manuscript. All authors contributed to the article and approved the submitted version.

## Funding

This research was supported by Qingdao Key Clinical Specialty Project Fund (QDZDZK-2022-097) and Qingdao Natural Science Foundation (23-2-1-201-zyyd-jch).

## Conflict of interest

LS is the director of BrainNow Medical Technology Limited and YL is now employed by BrainNow Medical Technology Limited.

The remaining authors declare that the research was conducted in the absence of any commercial or financial relationships that could be construed as a potential conflict of interest.

## Publisher’s note

All claims expressed in this article are solely those of the authors and do not necessarily represent those of their affiliated organizations, or those of the publisher, the editors and the reviewers. Any product that may be evaluated in this article, or claim that may be made by its manufacturer, is not guaranteed or endorsed by the publisher.
